# Implications of Malnutrition on Contrast-Associated Acute Kidney Injury in Young and Old Patients Undergoing Percutaneous Coronary Intervention: A Multicenter Prospective Cohort

**DOI:** 10.3389/fnut.2021.795068

**Published:** 2022-02-08

**Authors:** Jingjing Liang, Lingyu Zhang, Zhidong Huang, Yibo He, Yihang Ling, Kai Chen, Ming Ying, Mengfei Lin, Guode Li, Jin Liu, Yong Liu, Yan Liang, Shiqun Chen, Yunzhao Hu

**Affiliations:** ^1^Department of Cardiology, Shunde Hospital, Southern Medical University, Foshan, China; ^2^The Second School of Clinical Medicine, Southern Medical University, Guangzhou, China; ^3^Department of Cardiology, Maoming People's Hospital, Maoming, China; ^4^Department of Cardiology, Guangdong Provincial Key Laboratory of Coronary Heart Disease Prevention, Guangdong Cardiovascular Institute, Guangdong Provincial People's Hospital, Guangdong Academy of Medical Sciences, Guangzhou, China

**Keywords:** malnutrition, percutaneous coronary intervention, contrast-associated acute kidney injury, the controlling nutritional status score, 1-year mortality

## Abstract

**Background:**

The relationship between malnutrition and the risk of contrast-associated acute kidney injury (CA-AKI) and the resulting prognosis in patients undergoing percutaneous coronary intervention (PCI) is still not well known.

**Methods:**

Patients undergoing PCI were consecutively enrolled in a multicenter study in China (NCT01402232), categorized by nutritional status (non-malnutrition, malnutrition) based on two different cut-off values (i.e., traditional threshold and the best cut-off value based on the receiver operating characteristic (ROC) curve) for the controlling nutritional status (CONUT) score. The primary endpoint was CA-AKI, diagnosed as a rise in serum creatinine >0.3 mg/dl or >50% than the baseline level occurring within 48 h after the intervention. The secondary endpoint was all-cause mortality. The relationships of malnutrition, CA-AKI, and all-cause mortality were examined using multivariate-adjusted logistic and Cox regression analyses, respectively.

**Results:**

Among 2,083 patients undergoing PCI (age: 62.8 ± 11.1 years; 79.0% men), 1,258 (60.4%) were malnourished. During hospitalization, 80 (3.8%) patients developed CA-AKI events. The incidence of CA-AKI in patients who did not have malnutrition (the non-malnutrition group) and those who did have malnutrition (the malnutrition group) was 1.7% and 5.25%, respectively. Patients with malnutrition had a 2-fold increased adjusted risk of CA-AKI compared to those with no malnutrition [adjusted odds ratio (aOR) (95% confidence interval CI): 2.41 (1.22 to 5.22)]. Malnutrition was associated with a 3-fold increased adjusted risk of CA-AKI in patients aged ≤ 75 years [*N* = 1,791, aOR (95% CI): 3.39 (1.46–9.25)]. Malnourished patients with CA-AKI had a higher risk of all-cause mortality than the others. Similar results were observed in the grouping of Supplemental Analyses based on the optimal cut-off value of the CONUT score identified by the ROC curve.

**Conclusions:**

Malnutrition is strongly associated with an increased risk of CA-AKI in both young and old patients undergoing PCI. Malnourished patients with CA-AKI had a significantly higher risk of all-cause mortality. Further studies are needed to prospectively assess the efficacy of nutritional interventions on outcomes in patients undergoing PCI.

## Introduction

Contrast-associated acute kidney injury (CA-AKI) is a major complication of percutaneous coronary intervention (PCI) and is associated with poor prognosis (1, 2). The incidence of CA-AKI in patients undergoing PCI ranged from 6 to 18% (3–5). Due to limited CA-AKI treatment strategies, early screening and preventive measures for this high-risk population are essential.

Malnutrition is strongly associated with oxidative stress and the inflammatory process (6, 7). CA-AKI is highly related to neutrophils and albumin, which are well-known biomarkers of inflammation (8, 9), and is also affected by malnutrition. Protein-caloric malnutrition is related to kidney hemodynamic changes, the reduction of renal blood flow, glomerular filtration rate, and the ability of renal tubules to excrete acid (10, 11), which are involved in the physiological mechanisms that occur during CA-AKI.

Compared with other risk factors, malnutrition is easier to recognize and reverse by physicians (12). The controlling nutritional status (CONUT) score is an efficient and simple tool to detect malnutritional status, based on only three indexes (serum albumin, cholesterol, and lymphocytes), and has been widely used in the cardiovascular field (13, 14). Recent evidence has shown that malnutrition is highly prevalent in patients with cardiovascular diseases, increasing the risk of complications and adverse clinical outcomes (15–17). However, the relationship between malnutrition and the risk of CA-AKI and the resultant prognosis in patients undergoing PCI have not been adequately addressed.

Therefore, this study investigates the implications of malnutrition on CA-AKI and the resulting mortality among all patients undergoing PCI in a large multicenter cohort.

## Methods

### Study Population

**Figure 1:**
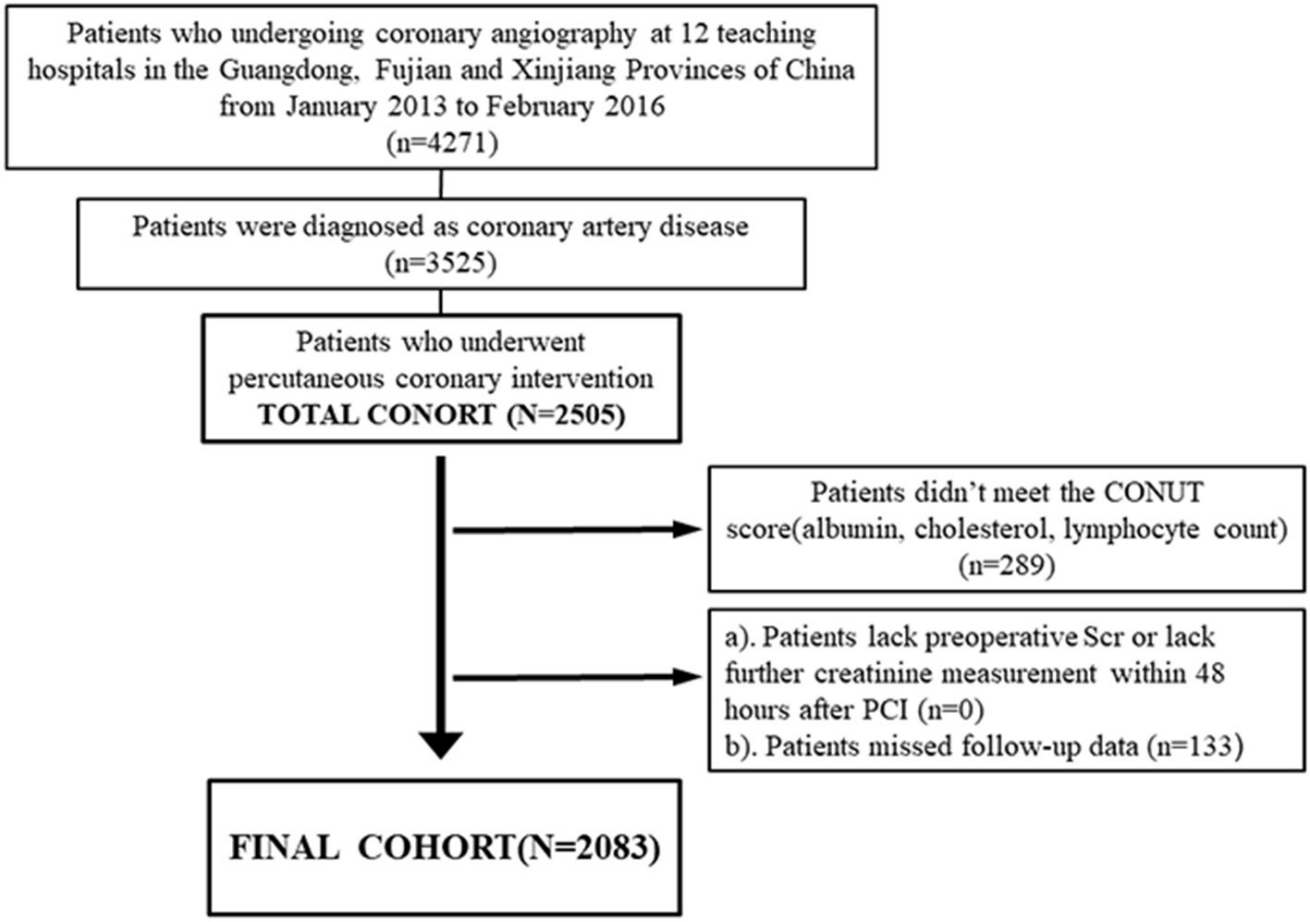
Patient flow diagram.

The REICIN study, a prospective, multicenter study (NCT01402232), enrolled a total of 4,271 patients from three different provinces of China, admitted to one of 12 hospitals between Jan 2013 and February 2016 and undergoing coronary angiography. Initially, all patients who underwent PCI with the diagnosis of coronary artery disease (CAD) were initially considered for inclusion. Patients corresponding to the following criteria were then excluded: (1) Patients who did not meet the CONUT score conditions (available serum albumin, lymphocyte, total cholesterol); (2) patients with missing follow-up data; (3) patients with a lack of preoperative serum creatinine or lack of further creatinine measurement within 48 h after PCI. Eventually, 2,083 patients were included in the final analysis (Figure 1). The ethics committee waived the requirement for written informed consent by participants because our study was retrospective in nature (No. GDREC2012141H). The date of approval by the ethics committee was 2011-11-19.

### Data Collection

The data from the primary and secondary care records were retrieved from electronic medical records from the hospitals, including demographic characteristics, laboratory data, comorbid conditions, and medical therapy. Follow-up information was collected from follow-up appointments, re-hospitalization records of the clinical management system, and conversations between the patients and their families and the attending physicians or well-trained research assistants, over the telephone. Serum creatinine (Scr) was measured at least twice, before PCI, and 1 to 2 days following the procedure. Baseline values of laboratory examinations (e.g., serum total cholesterol, albumin, and total lymphocyte counts) were obtained from preoperative blood sampling data.

### Malnutrition Screening Tools

The controlling nutritional status (CONUT) score was chosen as the malnutrition assessment method in our study because of its high applicability to cardiovascular disease (18). Based on the traditional threshold of the CONUT score, we divided the patients into two groups: non-malnutrition (scores of 0 to 1) and malnutrition (scores of 2 to 12). We added a Supplementary analysis to the attachment, using receiver operating characteristic (ROC) to determine the optimal cut-off value (2.50). The population was divided into two groups: CONUT ≤ 2 (lower risk of malnutrition, scores of 0 to 2) and CONUT >2 (higher risk of malnutrition, scores of 3 to 12).

### Endpoint and Definition

The primary endpoint was CA-AKI diagnosed as a rise in serum creatinine >0.3 mg/dl or >50% than the baseline level, occurring within 48 h after the intervention. The secondary endpoint was all-cause mortality. Kidney function was assessed by the estimated glomerular filtration rate (eGFR), which was calculated using the Modification of Diet in Renal Disease(MDRD) study equation. Chronic kidney disease (CKD) was defined as eGFR < 60 mL/min/1.73 m^2^ at baseline (19). Congestive heart failure (CHF) was diagnosed as Killip class > 1 or New York Heart Association class > 2.

### Statistical Analysis

Descriptive statistics were reported as mean ± standard deviation (SD) for continuous variables, and categorical variables were expressed as numbers (percentages) at baseline. Student *t*-tests and nonparametric tests (Mann–Whitney) were used to compare normally and not normally distributed variables, respectively. Differences in categorical variables were compared using the chi-square test. Multivariate logistic regression analysis was used to evaluate the association between malnutrition and CA-AKI, with or without adjustment for age, preoperative Scr, anemia, body mass index (BMI), contrast volume, CHF, intra-aortic balloon pump, and diuretics. Combined with the sample size, the confounding variables were associated with known risks of CA-AKI or mortality, based on previous studies and clinical plausibility, or a *p*-value of < 0.1, in univariable analysis.

The optimal cut-off value of the CONUT score was determined by the best performance based on the appropriate sensitivity and specificity ratio on the ROC curve. Time-to-event data were graphically presented using the Kaplan–Meier curves. The log-rank tests were used to compare survival between groups. Multivariable Cox regression models were used to estimate hazard ratios for all-cause mortality across combined nutritional statuses and the occurrence of CA-AKI, with adjustment for age, sex, CKD, and CHF.

All tests were two-sided and *P* values < 0.05, were considered statistically significant. All statistical analyses were performed using the R software (ver. 4.0.3).

## Results

### Baseline Characteristics

Among the 2,083 patients undergoing PCI, the mean age was 62.8 ± 11.1 years, 79% were men, and 258 (60.4%) were malnourished. Compared with the non-malnutrition group, malnourished patients were older, but there was no significant difference in the proportion of sex, diabetes mellitus (DM), and smoking. Malnourished patients had a higher prevalence of anemia, hypertension, CKD, CHF, and the use of diuretics, as well as higher Scr, but lower BMI. There were no significant differences between the two groups in contrast agent dose, statin use, and other preoperative medications. More data on baseline demographic and clinical characteristics of the patients are shown in Table 1. Similar trends of baseline characteristics based on the cut-off value (2.5) of the CONUT score are detailed in Supplemental Table 1.

**Table 1:** Baseline characteristics stratified by risk of malnutrition (Cut-off value based on a traditional threshold).

**Characteristics**	**Overall**	**Non-malnutrition**	**Malnutrition**	***p*** **-value**
	(*n* = 2,083)	(*n* = 825)	(*n* = 1,258)	
**Demographic characteristics**				
Age, year	62.81 (11.09)	60.0 ± 11.0	64.6 ± 10.7	<0.001
Male, *n* (%)	1,646 (79.02)	641 (77.70)	1,005 (79.89)	0.252
**Basic information**				
SBP, mmHg	131.12 (20.32)	133.59 ± 19.59	129.50 ± 20.64	<0.001
DBP, mmHg	76.31 (11.97)	77.79 ± 11.67	75.33 ± 12.07	<0.001
BMI, kg/m^2^	24.12 (3.27)	24.47 ± 3.30	23.88 ± 3.24	<0.001
**Medical history**				
Anemia, *n* (%)	653 (31.39)	145 (17.62)	508 (40.41)	<0.001
DM, *n* (%)	597 (28.66)	223 (27.03)	374 (29.73)	0.199
Hypertension, *n* (%)	1,171 (56.22)	431 (52.24)	740 (58.82)	0.004
Smoke, *n* (%)	899 (43.16)	364 (44.12)	535 (42.53)	0.501
CKD, *n* (%)	1,111 (53.34)	374 (45.33)	737 (58.59)	<0.001
CHF, *n* (%)	493 (23.67)	173 (20.97)	320 (25.44)	0.022
AMI, *n* (%)	792 (38.02)	281 (34.06)	511 (40.62)	0.003
IABP, *n* (%)	44 (2.11)	15 (1.82)	29 (2.31)	0.545
**Laboratory findings**				
Hemoglobin, g/L	133.90 (16.63)	138.74 ± 14.41	130.73 ± 17.23	<0.001
TC, mmol/L	4.59 (1.29)	5.18 ± 1.08	4.20 ± 1.26	<0.001
LYMPH, 10∧9/L	1.92 (0.97)	2.29 ± 1.18	1.68 ± 0.71	<0.001
ALB, g/L	36.55 (4.40)	38.96 ± 2.90	34.98 ± 4.50	<0.001
Scr, umol/L	85.00 [73.00, 104.00]	83.00 [72.00, 96.00]	88.00 [74.00, 108.00]	<0.001
eGFR, ml/mim/1.73 m^2^	80.30 [63.22, 95.24]	84.81 [69.07, 98.09]	77.04 [59.39, 92.74]	<0.001
TBIL, mg/dl	14.50 (6.62)	14.31 ± 6.28	14.63 ± 6.83	0.277
Uric acid, μmol/L	374.73 [315.00, 446.00]	379.00 [325.72, 446.00]	370.56 [309.00, 444.75]	0.053
CRP, mg/L	1.98 [0.00, 7.39]	1.73 [0.00, 5.58]	2.31 [0.00, 10.10]	0.029
Urine pro, g/L	0.25 [0.10, 0.70]	0.25 [0.20, 0.30]	0.25 [0.10, 0.70]	0.977
Contrast volume, ml	110.00 [100.00, 150.00]	100.00 [100.00, 150.00]	115.00 [100.00, 150.00]	0.171
**Treatment**				
Pre-statin, *n* (%)	1,346 (64.62)	545 (66.06)	801 (63.67)	0.286
Pre-CCB, *n* (%)	203 (9.75)	75 (9.09)	128 (10.17)	0.459
Pre-ACEI/ARB, *n* (%)	861 (41.33)	347 (42.06)	514 (40.86)	0.617
Pre-diuretics, *n* (%)	221 (10.61)	53 (6.42)	168 (13.35)	<0.001

*DM, diabetes mellitus; CKD, chronic kidney disease; CHF, congestive heart failure; AMI, acute myocardial infarction; IABP, intra-aortic ball on pump; SBP, systolic blood pressure; DBP, diastolic blood pressure; BMI, body mass index; TC, serum total cholesterol; ALB, albumin; Scr, serum creatinine; eGFR, estimated glomerular filtration rate; TBIL, serum total bilirubin; UA, uric acid; CRP, C-reactive protein; Urine pro, Urine protein; Pre-CCB, Pre-calcium channel blocker; Pre-ACEI/ARB, Pre-angiotensin-converting enzyme inhibitor/ angiotensin receptor blocker; CONUT score, Controlling Nutritional Status score*.

### Clinical Outcomes

**Figure 2:**
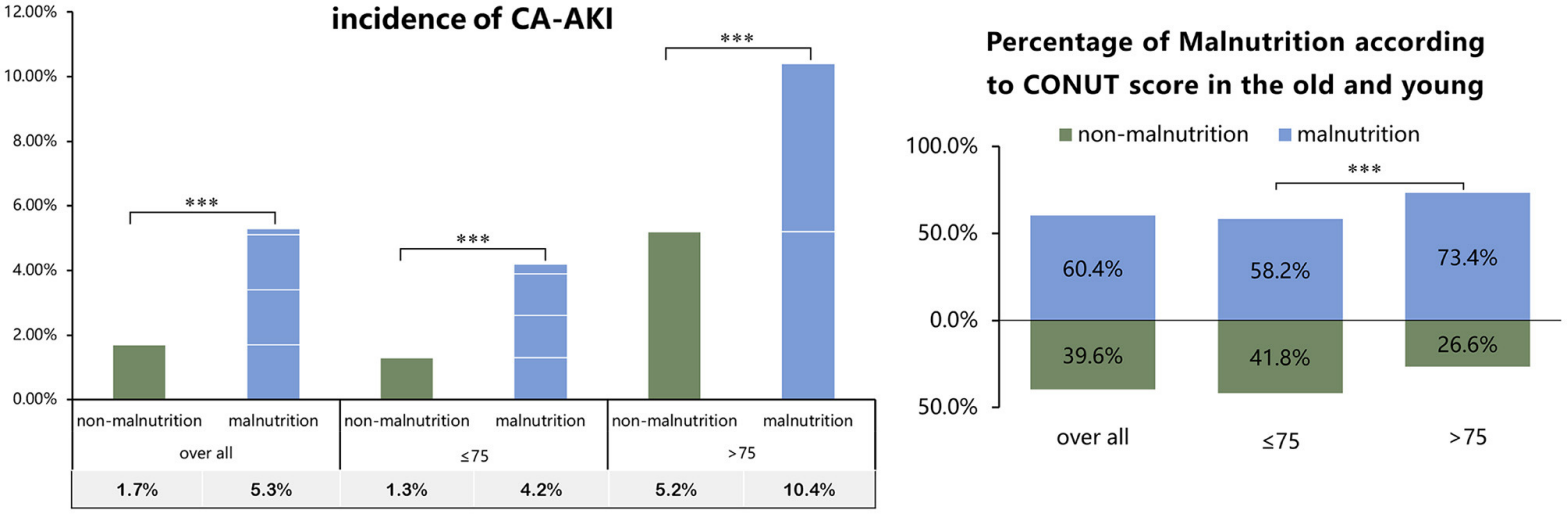
Percentage of contrast-associated acute kidney injury (CA-AKI) incidence and malnutrition in old and young patients with percutaneous coronary intervention (PCI) (Cut-off value based on traditional threshold). ***p < 0.001.

During the study period, 80 patients (3.8%) developed CA-AKI events. The incidence of CA-AKI in patients with and without malnutrition was 1.7 and 5.3%, respectively (*p* < 0.001), and malnutrition was observed among patients aged ≤ 75 years (58.2%) and those aged >75 years (73.4%) (Figure 2). The incidence of CA-AKI, graded by age and nutritional status, is shown in Figure 2. Controlling potential confounding variables, malnutrition was associated with a 2-fold increased risk of CA-AKI in patients with PCI [adjusted odds ratio (95% confidence interval), aOR (95% CI): 2.41(1.22–5.22)] (Table 2). It is worth noting that malnutrition was associated with a 3-fold increase in the risk of CA-AKI in patients undergoing PCI with age ≤ 75 years in the multivariate-adjusted logistic regression model [N = 1,791, aOR (95% CI): 3.39 (1.46–9.25)] (Table 3). We also observed a higher risk of CA-AKI in patients with malnutrition based on the cut-off value (2.5) of the CONUT score (Supplemental Tables 2, 3). Kaplan–Meier curves for all-cause mortality across nutritional statuses and the occurrence of CA-AKI are shown in Supplemental Figure 2. In the Cox model, malnourished patients with or without CA-AKI had a significantly higher risk of all-cause mortality after adjusting for confounding factors. Patients with malnutrition and CA-AKI had the highest risk of mortality [adjusted hazard ratio (95% CI), aHR (95% CI): 4.1(2.17–7.75)]. Similar results are reported in Supplemental Table 4, using the optimal cut-off value determined by ROC.

**Table 2:** Risk of contrast-associated acute kidney injury (CA-AKI)-associated nutritional state in patients undergoing percutaneous coronary intervention (PCI) (Cut-off value based on a traditional threshold).

	**Univariate**		**Multivariate**	
**Risk factors**	**OR (95% CI)**	***p*** **-value**	**OR (95% CI)**	***p*** **-value**
Non-malnutrition	Ref		Ref	
Malnutrition	3.207 (1.846–5.984)	<0.0001	2.408 (1.218–5.215)	0.017
Age, year			1.048 1.018–1.080)	0.002
Scr, umol/L			1.006 (1.000–1.011)	0.025
Anemia			1.032 (0.565–1.846)	0.916
BMI, kg/m			1.027 (0.939–1.120)	0.555
Contrast volume, ml			1.001 (0.995–1.005)	0.764
CHF			0.728 (0.348–1.423)	0.374
IABP			8.090 (2.367–23.937)	<0.0001
Pre-diuretics			2.007 (0.964–3.941)	0.051

^*^Odds ratio adjusted for age, Scr, serum creatinine; Anemia, HGB, hemoglobin; BMI, body mass index; contrast volume, CHF, congestive heart failure; IABP, intra-aorticball on pump; and pre-diuretics.

**Table 3:** Risk of CA-AKI-associated nutritional state in patients undergoing PCI with age ≤ 75 years (N = 1791) (Cut-off value based on traditional threshold).

	**Univariate**		**Multivariate**	
**Risk factors**	**OR (95% CI)**	***p*** **-value**	**OR (95% CI)**	***p*** **-value**
Non-malnutrition	Ref		Ref	
Malnutrition	3.250 (1.694–6.881)	0.001	3.386 (1.458–9.253)	0.008
Age, year			1.021 (0.982–1.064)	0.319
Scr, umol/L			1.005 (0.998–1.011)	0.094
Anemia			0.881 (0.399–1.844)	0.743
BMI, kg/m			1.022 (0.916–1.136)	0.689
Contrast volume, ml			1.003 (0.997–1.008)	0.338
CHF			0.675 (0.256–1.567)	0.390
IABP			8.451 (1.762–30.502)	0.002
Pre-diuretics			1.701 (0.592–4.190)	0.280

^*^Odds ratio adjusted for age, Scr, serum creatinine; Anemia, HGB, hemoglobin; BMI, body mass index; contrast volume, CHF, congestive heart failure; IABP, intra-aortic ball on pump; and pre-diuretics.

## Discussion

This was a national prospective multicenter cohort study to demonstrate the association between malnutrition and CA-AKI in patients undergoing PCI. Compared with no malnutrition, we found that malnutrition was significantly associated with a 2-fold increased risk of developing CA-AKI. The incidence of CA-AKI was higher in elderly patients aged >75 years, while the malnutrition-associated risk of CA-AKI in younger patients aged ≤ 75 years appeared to be higher. Malnourished patients with CA-AKI had a higher risk of all-cause mortality.

Malnutrition is prevalent in all patients undergoing PCI. In our study, nearly 60% of the patients were defined as malnourished by the CONUT score. Malnutrition is prevalent in both young and elderly PCI patients and is more common in older patients. Few studies have reported the prevalence of malnutrition in younger patients undergoing PCI, and our findings are in line with the available evidence. Xiao et al. have shown that 61.1% of patients undergoing PCI were malnourished, using the CONUT score in 1,308 patients aged ≥75 years (20). Sergio et al. found malnutrition in 50 to 60% of patients, evaluated by CONUT and nutritional risk index (NRI) scores, in a cohort of 6,023 patients with the acute coronary syndrome (ACS) (18). This study showed a considerable number of malnourished patients who underwent PCI. However, malnutrition is not seriously considered as a factor by clinical cardiologists. Patients who are admitted for PCI need to be screened for nutritional status and receive timely diagnoses and treatments. The indexes in the CONUT score used in this study are simple for clinicians to obtain and calculate.

We confirmed that malnutrition was strongly associated with an increased risk of CA-AKI, as assessed by the CONUT score. Previous studies have shown that malnutrition is an important risk factor for acute kidney injury (AKI). Recently, Acarbaş et al. reported that preoperative malnutrition, assessed using the prognostic nutritional index (PNI), CONUT score, and geriatric NRI (GNRI), was a predictor for AKI in a cohort of 454 patients (21). Miyeun et al. revealed that patients with a low PNI had an independent association with CA-AKI (22). The malnutrition index analyzed in our study focused on the CONUT score. The CONUT score included only three laboratory values (serum albumin, total cholesterol levels, and total lymphocyte count), and the assessed nutritional state was positively correlated with these three values (13). These laboratory indexes have previously been demonstrated as risk factors for CA-AKI. Decreased albumin levels were independently associated with an increased risk of CA-AKI in a cohort of 394 patients undergoing PCI (23). Lymphocyte count is an independent risk factor for the development of contrast-induced nephropathy (24). Qin et al. showed that total cholesterol was significantly higher in patients with CA-AKI than in non-CA-AKI patients (25). The rationale behind these associations may be explained by the main mechanisms of vasoconstriction and oxidative stress in CA-AKI.

The potential mechanisms underlying the relationship between malnutrition and CA-AKI include the role of proteins, lipid profiles, and lymphocytes. Albumin not only reflects the nutritional status of the patients but also influences microvascular integrity and participates in the inflammatory pathways (26). A lower serum albumin level is an independent prognostic predictor of several cardiovascular diseases, such as ACS, coronary artery disease (CAD), and heart failure (27, 28). Low levels of albumin may be involved in the development of CA-AKI through the vascular and oxidative inflammatory pathways. Lymphocytes, markers of inflammatory response and immune status, have been shown to participate in the initiation, proliferation, and recovery stages of AKI in the previous study (29). Lipid profiles have long been regarded as risk factors for cardiovascular and kidney diseases (30, 31). Previous studies have reported that low cholesterol levels might be related to predisposing catabolic comorbidity (32, 33), thus exacerbating metabolic disorders in CA-AKI. Failure to identify and intervene in malnourished patients before surgery may lead to the unnecessary occurrence of CA-AKI. Therefore, CA-AKI can be prematurely prevented by screening for malnutrition.

It is also worth mentioning that patients aged ≤ 75 years have a significantly high risk of CA-AKI in malnourished nutritional status. Compared with patients without malnutrition, patients with worse nutritional status aged ≤ 75 years showed a 3-fold increase in the incidence of CA-AKI, while those aged >75 years showed a 2-fold increase. This suggests that younger patients are more susceptible to nutritional status. Few studies have analyzed the relationship between malnutrition and CA-AKI in patients aged ≤ 75 years undergoing PCI. Wei et al. showed that malnutrition was an independent risk factor for CA-AKI in a cohort of 1,308 elderly patients aged >75 years undergoing PCI, based on the CONUT score (20). In our study, patients aged ≤ 75 years constituted a large proportion of the overall PCI population. Nutrition screening on admission and subsequent treatments should be performed in patients with PCI regardless of age, in the elderly and young.

Malnutrition increased the risk of mortality in patients who underwent PCI with or without CA-AKI. Malnourished patients with CA-AKI had the strongest association with all-cause mortality. Recent studies have shown that malnutrition was a significant risk factor of all-cause death in patients with CAD (34). Patients with CA-AKI were more likely to have adverse outcomes than those without CA-AKI (35). Malnutrition and kidney damage due to exposure to contrast media affect physical regulation and repair, which are related to adverse outcomes (36, 37).

All our findings strongly support that malnutrition is a potentially modifiable risk, a therapeutic target, and physicians should add screening for malnutrition in their daily practice. Early diagnosis of malnutrition may be associated with CA-AKI risk stratification, provide a warning sign, and guide clinicians to adopt secondary prevention measures for patients. Preoperative malnutrition assessment is difficult in patients undergoing PCI because of the limited time in an emergency. However, the CONUT score is easy to calculate for clinicians to effectively identify poor nutritional status. Screening patients who underwent PCI for malnutrition might help to identify the risk of CA-AKI, and these patients might benefit from tailoring dehydration prevention programs and nutritional supplements to prevent CA-AKI and ameliorate the prognosis. Intravenous hydration is a common strategy used by physicians to prevent CA-AKI (38). Increased hydration volume could accelerate the excretion of the contrast agent, decrease the release of vasoconstrictors and reactive oxygen species, and reduce direct renal toxicity (39). To reduce the residual risk and improve the prognosis, cardiologists should keep pace with current evidence and follow the nutrition guidelines in advance. Multiple strategies have been advocated to prevent and intervene in malnutrition, including dietary counseling, exercise standards, oral nutritional supplements, and educational interventions. Moreover, these nutritional interventions need to be performed during hospitalization and maintenance therapy after discharge. More rigorous research is needed to evaluate the efficiency of malnutrition intervention in patients with CA-AKI and mortality.

## Limitations

First, this was an observational study with a cross-sectional nature, so our inferences did not reflect direct causality. In addition, we must always recognize the potential for residual, uncontrolled confounding, which might partly explain the associations. Second, we did not have information about the marital status, educational attainment, or socio-economic information that might help us to apply more comprehensive nutritional assessments to identify the nutritional status. The complexity of malnutrition, especially in fatter or older adults, might be explained by the wide range of determinants and the diversity in etiology. Lastly, due to the lack of other endpoints of mortality, we could have missed some other points to predict the prognosis of patients who underwent PCI. We welcome other researchers and other countries with different social systems and healthcare to confirm our findings.

## Conclusion

In all patients undergoing PCI, malnutrition significantly increased the risk of developing CA-AKI. Malnourished patients with CA-AKI had a significantly higher risk of all-cause mortality. Clinicians must evaluate and monitor the nutritional levels of patients undergoing PCI early. Further studies are needed to prospectively assess the efficacy of nutritional interventions on outcomes in patients undergoing PCI.

## Data Availability Statement

The raw data supporting the conclusions of this article will be made available by the authors, without undue reservation.

## Ethics Statement

Written informed consent was obtained from the individual(s) for the publication of any potentially identifiable images or data included in this article.

## Author Contributions

YHu, SC, JLia, and LZ provided research idea and study design. JLia, ZH, YHe, YLin, KC, MY, ML, GL, YLia, and YLiu handled the data acquisition. JLiu and SC took care of data analysis and interpretation. ZH, JLia, and JLiu handled the statistical analysis. YHu, SC, and LZ done the supervision, writing guidance, and mentorship. Each author contributed important intellectual content during manuscript drafting or revision and accepts accountability for the overall work by ensuring that questions on the accuracy or integrity of any portion of the work are appropriately investigated and resolved. All authors read and approved the final version.

## Funding

This research was supported by the Science and Technology Innovation Project from Foshan, Guangdong (FS0AA-KJ218-1301-0010), a multi-center study on key techniques for prevention, diagnosis and treatment of high risk coronary artery disease (DFJH2020026), and a Study on the function and mechanism of the potential target for early warning of the cardiorenal syndrome after acute myocardial infarction based on transformism (DFJH201919).

## Conflict of Interest

The authors declare that the research was conducted in the absence of any commercial or financial relationships that could be construed as a potential conflict of interest.

## Publisher's Note

All claims expressed in this article are solely those of the authors and do not necessarily represent those of their affiliated organizations, or those of the publisher, the editors and the reviewers. Any product that may be evaluated in this article, or claim that may be made by its manufacturer, is not guaranteed or endorsed by the publisher.
