# Supplementary material for: Implications of Malnutrition on Contrast-Associated Acute Kidney Injury in Young and Old Patients Undergoing Percutaneous Coronary Intervention: A Multicenter Prospective Cohort
Source: Front Nutr. 2022 Feb 8;8:795068. doi: 10.3389/fnut.2021.795068 (PMC8861456; doi:10.3389/fnut.2021.795068)
Supplement: Supplementary file 1 [file Data_Sheet_1.docx]

**The implications of malnutrition on contrast-associated acute kidney injury in young and old patients undergoing percutaneous coronary intervention: a multicenter prospective cohort**

*Liang et al*

**Supplemental Figure 1 The optimal cut-off** **determined by the ROC analysis**


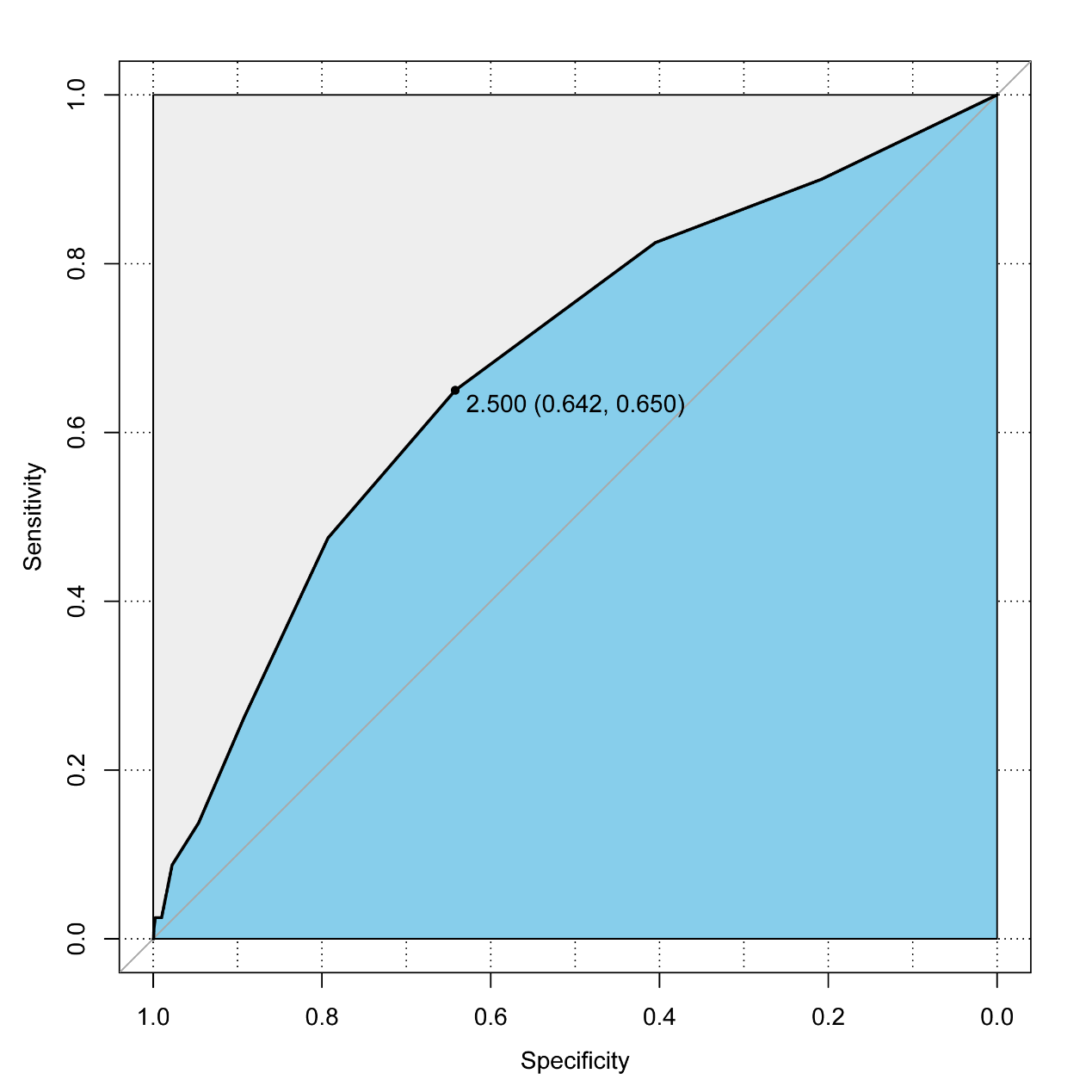


2.50 was detected as the best cut-off value with specificity=0.642 and sensitivity=0.650

**Supplemental Table 1 Baseline Characteristics stratified by risk of malnutrition (Cut-off value based on ROC curve)**

| **Characteristics** | **Overall** | **CONUT≤2** | **CONUT>2** | **p value** |
| --- | --- | --- | --- | --- |
|  | (n=2083) | (n=825) | (n=1258) |  |
| **Demographic characteristics** | | | | |
| Age, year | 62.81 (11.09) | 60.8 ±11.1 | 66.3 ±10.2 | <0.001 |
| Male, n (%) | 1646 (79.02) | 1035 (78.77) | 611 (79.45) | 0.752 |
| **Basic information** | | | | |
| SBP, mmHg | 131.12 (20.32) | 132.22±19.76 | 129.23±21.12 | 0.001 |
| DBP, mmHg | 76.31 (11.97) | 77.05±11.68 | 75.03±12.36 | <0.001 |
| BMI, kg/m^2^ | 24.12 (3.27) | 24.41±3.24 | 23.57±3.27 | <0.001 |
| **Medical history** | | | | |
| Anemia, n (%) | 653 (31.39) | 286 (21.80) | 367 (47.79) | <0.001 |
| DM, n (%) | 597 (28.66) | 361 (27.47) | 236 (30.69) | 0.129 |
| Hypertension, n (%) | 1171 (56.22) | 713 (54.26) | 458 (59.56) | 0.021 |
| Smoke, n (%) | 899 (43.16) | 563 (42.85) | 336 (43.69) | 0.741 |
| CKD, n (%) | 1111 (53.34) | 621 (47.26) | 490 (63.72) | <0.001 |
| CHF, n (%) | 493 (23.67) | 271 (20.62) | 222 (28.87) | <0.001 |
| AMI, n (%) | 792 (38.02) | 444 (33.79) | 348 (45.25) | <0.001 |
| IABP, n (%) | 44 (2.11) | 18 (1.37) | 26 (3.39) | 0.003 |
| **Laboratory findings** | | | | |
| Hemoglobin, g/L | 133.90 (16.63) | 137.47±15.02 | 127.80±17.47 | <0.001 |
| TC, mmol/L | 4.59 (1.29) | 4.90±1.20 | 4.05±1.25 | <0.001 |
| LYMPH, 10^9/L | 1.92 (0.97) | 2.15±1.04 | 1.53±0.70 | <0.001 |
| ALB, g/L | 36.55 (4.40) | 38.35±3.32 | 33.48±4.30 | <0.001 |
| Scr, umol/L | 85.00 [73.00, 104.00] | 83.25 [72.00, 98.00] | 90.00 [77.10, 113.00] | <0.001 |
| eGFR, ml/mim/1.73m^2^ | 80.30 [63.22, 95.24] | 83.67 [67.50, 97.15] | 73.83 [56.89, 89.79] | <0.001 |
| TBIL, mg/dL | 14.50 (6.62) | 14.35±6.23 | 14.76±7.23 | 0.17 |
| UA, mmol/L | 374.73 [315.00, 446.00] | 378.00 [320.50, 444.00] | 369.00 [305.00, 450.60] | 0.183 |
| CRP, mg/L | 1.98 [0.00, 7.39] | 1.58 [0.00, 5.57] | 3.98 [0.00, 14.62] | <0.001 |
| Urine pro, g/L | 0.25 [0.10, 0.70] | 0.25 [0.20, 0.50] | 0.25 [0.10, 0.70] | 0.591 |
| Contrast volume, ml | 110.00 [100.00, 150.00] | 100.00 [100.00, 150.00] | 120.00 [100.00, 150.00] | 0.409 |
| **Treatment** | | | | |
| Pre-statin, n (%) | 1346 (64.62) | 873 (66.44) | 473 (61.51) | 0.026 |
| Pre-CCB, n (%) | 203 (9.75) | 135 (10.27) | 68 (8.84) | 0.324 |
| Pre-ACEI/ARB, n (%) | 861 (41.33) | 555 (42.24) | 306 (39.79) | 0.295 |
| Pre-diuretics, n (%) | 221 (10.61) | 93 (7.08) | 128 (16.64) | <0.001 |

Abbreviation: DM, diabetes mellitus; CKD, chronic kidney disease; CHF, congestive heart failure; AMI, acute myocardial infarction; IABP, intra-aortic ball on pump; SBP, systolic blood pressure; DBP, diastolic blood pressure; BMI, body mass index; TC, serum total cholesterol; ALB, Albumin; Scr, serum creatinine; eGFR, estimated glomerular filtration rate; TBIL, serum total bilirubin; UA, Uric Acid; CRP, C-reactive protein; Urine pro, Urine protein; Pre-CCB, Pre-calcium channel blocker; Pre-ACEI/ARB, Pre-angiotensin-converting enzyme inhibitor/ angiotensin receptor blocker; CONUT score, Controlling Nutritional Status

**Supplemental Table 2 Multivariate risk of CA-AKI associated nutritional state in patients undergoing PCI (Cut-off value based on ROC curve)**

| Categories | OR (95%CI) | P value |
| --- | --- | --- |
| CONUT≤2 | Ref |  |
| CONUT>2 | 2.20(1.22-4.02) | 0.009 |
| Age, year | 1.05(1.02-1.08) | 0.003 |
| Scr, umol/L | 1.01(1.00-1.01) | 0.027 |
| Anemia | 1.00(0.54-1.82) | 0.997 |
| BMI,kg/m | 1.03(0.94-1.12) | 0.558 |
| Contrast volume, ml | 1.00(1.00-1.01) | 0.717 |
| CHF | 0.72(0.35-1.41) | 0.363 |
| IABP | 7.13(2.09-20.93) | 0.001 |
| Pre-diuretics | 1.91(0.92-3.76) | 0.070 |

*Odds ratio adjusted for age, serum creatinine (Scr), Anemia, hemoglobin (HGB), body mass index(BMI) , contrast volume, congestive heart failure (CHF), intra-aortic ball on pump (IABP)，pre-diuretics.

**Supplemental Table 3 Multivariate risk of CA-AKI associated nutritional state in patients undergoing PCI with age ≤75 year (N=1791)** **(Cut-off value based on ROC curve)**

| Categories | OR (95%CI) | P value |
| --- | --- | --- |
| CONUT≤2 | Ref |  |
| CONUT>2 | 2.98(1.46-6.24) | 0.003 |
| Age, year | 1.02(0.98-1.06) | 0.359 |
| Scr, umol/L | 1.01(1.00-1.01) | 0.112 |
| Anemia | 0.84(0.37-1.77) | 0.648 |
| BMI,kg/m | 1.03(0.92-1.14) | 0.651 |
| Contrast volume, ml | 1.00(1.00-1.01) | 0.340 |
| CHF | 0.67(0.26-1.55) | 0.379 |
| IABP | 7.14(1.52-5.26) | 0.005 |
| Pre-diuretics | 1.62(0.56-4.01) | 0.330 |

*Odds ratio adjusted for age, serum creatinine (Scr), Anemia, hemoglobin (HGB), body mass index(BMI) , contrast volume, congestive heart failure (CHF), intra-aortic ball on pump (IABP)，pre-diuretics.

**Supplemental Figure 2 Kaplan-Meier curves for all-cause mortality across CA-AKI incidence and nutrition state groups of study participants in different cut-off values**


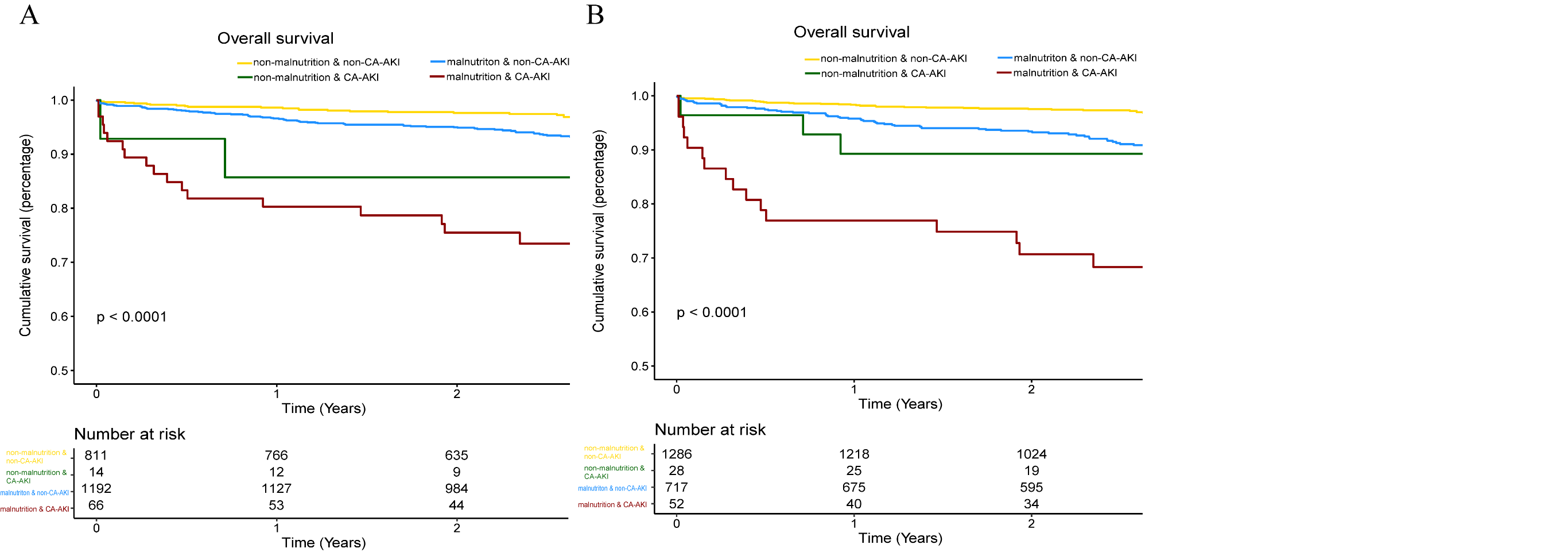


Kaplan-Meier curves for all-cause mortality by the Controlling Nutritional Status score (CONUT score) and the incidence of CA-AKI. (A) The CONUT score (Cut-off value based on traditional threshold): 0 to 1 reflects normal and 2 to 12 reflects malnutrition. (B) The CONUT score (Cut-off value based on ROC curve): 0 to 2 reflects normal and 3 to 12 reflects malnutrition.

**Supplemental Table 4 Risk of all-cause mortality across nutrition state and CA-AKI incidence**

|  | Model 1^a^ | |  | Model 2^b^ | |
| --- | --- | --- | --- | --- | --- |
| Categories | HR (95%CI) | P value |  | HR (95%CI) | P value |
| non-malnutrition and non-CA-AKI | Ref |  |  | Ref |  |
| non-malnutrition and CA-AKI | 3.58(0.84-15.17) | 0.084 |  | 2.14(0.66-7.00) | 0.207 |
| malnutrition and non-CA-AKI | 1.61(1.04-2.49) | 0.034 |  | 2.06(1.40-3.03) | <0.001 |
| malnutrition and CA-AKI | 4.10(2.17-7.75) | <0.001 |  | 5.36(2.95-9.75) | <0.001 |
| Age, year | 1.06(1.04-1.08) | <0.001 |  | 1.06(1.04-1.08) | <0.001 |
| gender | 1.00(0.67-1.49) | >0.99 |  | 0.98(0.66-1.47) | 0.931 |
| CKD | 1.22(0.83-1.82) | 0.314 |  | 1.19(0.80-1.77) | 0.381 |
| CHF | 2.14(1.51-3.03) | <0.001 |  | 2.08(1.47-2.94) | <0.001 |

*Hazard ratio adjusted for age, gender, chronic kidney disease (CKD), congestive heart failure (CHF).

^a^ Criteria for malnutrition classification (Cut-off value based on traditional threshold): non-malnutrition (scores of 0, 1), malnutrition (scores of 2 to 12).

^b^ Criteria for malnutrition classification (Cut-off value based on ROC curve): CONUT≤2 (scores of 0 to 2), CONUT>2 (scores of 3 to 12).
